# The Nutritional Challenges Following Revisional Bariatric Surgery After Sleeve Gastrectomy: A Systematic Review and Meta Analysis

**DOI:** 10.1007/s11695-025-08325-2

**Published:** 2025-11-05

**Authors:** Hassan El-Masry, Mohamed H. Mahmoud, Batool Sami Mohamed, Dina Elraggal, Mostafa H. Elkholy, Ahmed Abokhozima

**Affiliations:** 1 Faculty of Medicine, Alexandria University, Alexandria, Egypt; 2 MIAX Research Lab, Alexandria, Egypt

**Keywords:** Revisional bariatric surgery, Failed sleeve gastrectomy, Micronutrient, Malnutrition, Vitamin deficiency

## Abstract

**Background:**

Laparoscopic sleeve gastrectomy (LSG) is one of the most commonly performed metabolic and bariatric surgeries (MBS) worldwide due to its simplicity and efficacy. However, long-term outcomes have revealed limitations, including recurrent weight regain, inadequate weight loss, and nutritional deficiencies, leading to an increasing need for revisional bariatric surgery. While revisional procedures can restore weight loss efficacy, they may also carry additional risks of micronutrient and protein deficiencies due to anatomical and physiological alterations.

**Methods:**

This review was conducted in accordance with PRISMA guidelines and included studies published up to April 2025. A systematic search of PubMed, Scopus, Web of Science, and Cochrane Library was performed. Eligible studies reported nutritional outcomes after revisional bariatric procedures following LSG. Data on vitamin D, vitamin B12, iron, calcium, albumin levels, and anemia were extracted. Meta-analyses were conducted for both continuous and categorical outcomes.

**Results:**

Fourteen studies with a total of 1,049 patients were included. Deficiencies in key micronutrients such as vitamin D, vitamin B12, albumin, calcium, zinc and iron were reported across revision types. A co-author network analysis was performed to identify and address overlapping cohorts. The analysis revealed notable rates of nutritional deficiencies across all procedures. Vitamin D deficiency was more frequent in certain revision surgeries. Iron deficiency showed statistically significant differences between groups (*p* = 0.025), with one revision type associated with a higher risk (*p* = 0.0106). Albumin and anemia rates did not differ significantly between procedures, but anemia remained a common complication overall.

**Conclusion:**

Micronutrient deficiencies are notably prevalent following revisional bariatric surgery after LSG. Despite the routine use of supplementation regimens, deficiencies in vitamin D, vitamin B12, iron, and protein continue to be frequently reported. Considerable variability among surgical techniques highlights the need for individualized nutritional monitoring and the implementation of standardized supplementation protocols.

**Supplementary Information:**

The online version contains supplementary material available at 10.1007/s11695-025-08325-2.

## Introduction

Laparoscopic sleeve gastrectomy (LSG) has emerged as one of the most commonly performed metabolic and bariatric surgeries (MBS) globally, owing to its technical simplicity, efficacy in weight reduction, and substantial improvement in obesity-related comorbidities, including type 2 diabetes and hypertension [1–3]. The procedure involves resection of approximately 80% of the stomach, resulting in both restrictive and hormonal effects that promote satiety and metabolic benefits through modulation of gut hormones such as ghrelin [4].

Despite these advantages, accumulating evidence has revealed several long-term limitations of LSG. These include significant recurrent weight regain, development of de novo or worsening gastroesophageal reflux disease (GERD), inadequate weight loss in a subset of patients, and progressive nutritional deficiencies [5, 6]. Such complications have led to an increasing need for revisional bariatric surgery, with current estimates suggesting 15–20% of LSG patients may require secondary procedures [7, 8].

Revisional surgery following LSG presents unique technical challenges and heightened clinical risks compared to primary procedures. The altered anatomy, including sleeve dilation, strictures, and fibrotic changes along previous staple lines, contributes to increased operative complexity [9, 10]. Furthermore, revisional procedures are associated with substantially higher rates of postoperative complications, including anastomotic leaks (4.6% vs 0.45% in primary cases), surgical site infections, and bleeding [11].

From a nutritional perspective, conversion to malabsorptive procedures such as Roux-en-Y gastric bypass (RYGB) or single anastomosis duodeno-ileal bypass with sleeve (SADI-S) introduces additional concerns. These include exacerbated risks of micronutrient deficiencies (particularly iron, vitamin B12, vitamin D, and calcium) and protein-energy malnutrition, which may be more common in revisional cases than after primary LSG [12]. This systematic review aimed to evaluate the nutritional outcomes following revisional bariatric surgeries performed after LSG, with a particular focus on frequently reported micronutrients such as vitamin D, vitamin B12, iron, ferritin, calcium, albumin, and zinc, as well as protein-energy malnutrition.

## Methods

This systematic review and meta-analysis adhered to the Preferred Reporting Items for Systematic Reviews and Meta-Analyses (PRISMA) guidelines and the Cochrane Handbook for Systematic Reviews of Interventions [8, 9], and was prospectively registered in the PROSPERO database (CRD420251063133).

### Search Strategy

A comprehensive literature search was conducted across four major electronic databases: PubMed, Cochrane Library, Web of Science (WoS), and Scopus. The search included studies published up to April 10, 2025. The following combination of keywords and Boolean operators was used: (revision OR "revisional bariatric surgery" OR "reoperative bariatric surgery" OR "conversional bariatric surgery" OR reoperation OR "revision surgery" OR "post sleeve") AND ("sleeve gastrectomy" OR "vertical sleeve gastrectomy" OR "laparoscopic sleeve gastrectomy" OR LSG OR VSG OR "gastric sleeve" OR sleeve) AND (nutrition OR "nutritional deficiency" OR "micronutrient deficiency" OR "vitamin deficiency" OR "mineral deficiency" OR malnutrition OR "nutritional status" OR "nutritional complication"). Additional sources were identified by manually screening the references of relevant systematic reviews and meta-analyses.

### Inclusion and Exclusion Criteria

Eligible studies included randomized controlled trials (RCTs), cohort studies, case–control studies, and large case series involving ten or more patients. Systematic reviews and meta-analyses were also screened for relevant references. The target population consisted of adult patients who had previously undergone sleeve gastrectomy and required revisional bariatric surgery due to inadequate weight loss, complications, or nutritional concerns. Studies were included if they reported at least one nutritional outcome, such as deficiencies in vitamin D, vitamin B12, iron, or protein, or documented measures like albumin levels. Only studies published in the past 10 to 15 years were considered, and both English and non-English studies were eligible if a full translatable text was available.

Studies were excluded if they were case reports, editorials, conference abstracts without full data, or animal studies. Also excluded were those involving pediatric populations, primary bariatric procedures without prior sleeve gastrectomy, or non-surgical interventions. Endoscopic-only revisions were not included unless performed alongside surgical revisions. Additionally, studies that did not provide quantitative nutritional data, focusing solely on weight loss without relevant nutritional data, were excluded.

### Study Selection Process

The screening of studies was conducted in two stages. First, the titles and abstracts of the identified studies were screened for relevance. In the second stage, the full texts of potentially relevant studies were assessed for eligibility.

### Data Extraction

Four reviewers performed data extraction independently using a standardized data extraction form. The following information was extracted from each study: author, publication year, study design, sample size, surgical procedure, follow-up duration, average age of participants, and BMI values. Nutritional outcomes were collected using a data extraction form, and all data were reviewed by the fifth reviewer. Any discrepancies between the reviewers were resolved through discussion and consensus.

### Quality Assessment

The quality assessment focused on key aspects such as study design, sample representativeness, comparability, outcome assessment, and follow-up. Randomized clinical trials were evaluated using the Cochrane Risk of Bias 2 (RoB 2) tool [13], while cohort and case–control studies were assessed using the Newcastle–Ottawa Scale (NOS) [14]. This approach ensured an appropriate and systematic appraisal tailored to each study design.

### Statistical Analysis

Single-arm meta-analyses were conducted using the meta package in R software (version 4.4.2) [15]. Heterogeneity among included studies was assessed using Chi-square and I-square statistical tests for continuous outcomes, pooled means and 95% confidence intervals (CIs) were calculated, while for dichotomous outcomes, pooled proportions and corresponding 95% CIs were computed. We considered homogeneity among studies when *P* > 0.1, I2 < 50%, and a fixed-effects model was chosen for meta-analysis; otherwise, a random-effect model was used, and when the heterogeneity persisted, we performed a sensitivity analysis. A subgroup meta-analysis was conducted to compare different revisional surgeries.

## Results

### Search Results

The initial search yielded a total of 653 studies from database sources, including 200 from PubMed, 284 from Scopus, 151 from Web of Science, and 18 from Cochrane. After removing 174 duplicate records, 479 unique studies remained for primary screening. Following the title and abstract screening, 436 studies were excluded for reasons such as being review articles (*n* = 28), being letters and editorials (*n* = 19), or being irrelevant. This left 39 full-text articles for detailed assessment. Of these, 30 were excluded for various reasons. 6 studies were included after a manual search of previous systematic reviews. Ultimately, 15 studies were included in the systematic review (supplementary appendix) [16–27, 29, 30, 31].

### Systematic Review

**Fig. 1:**
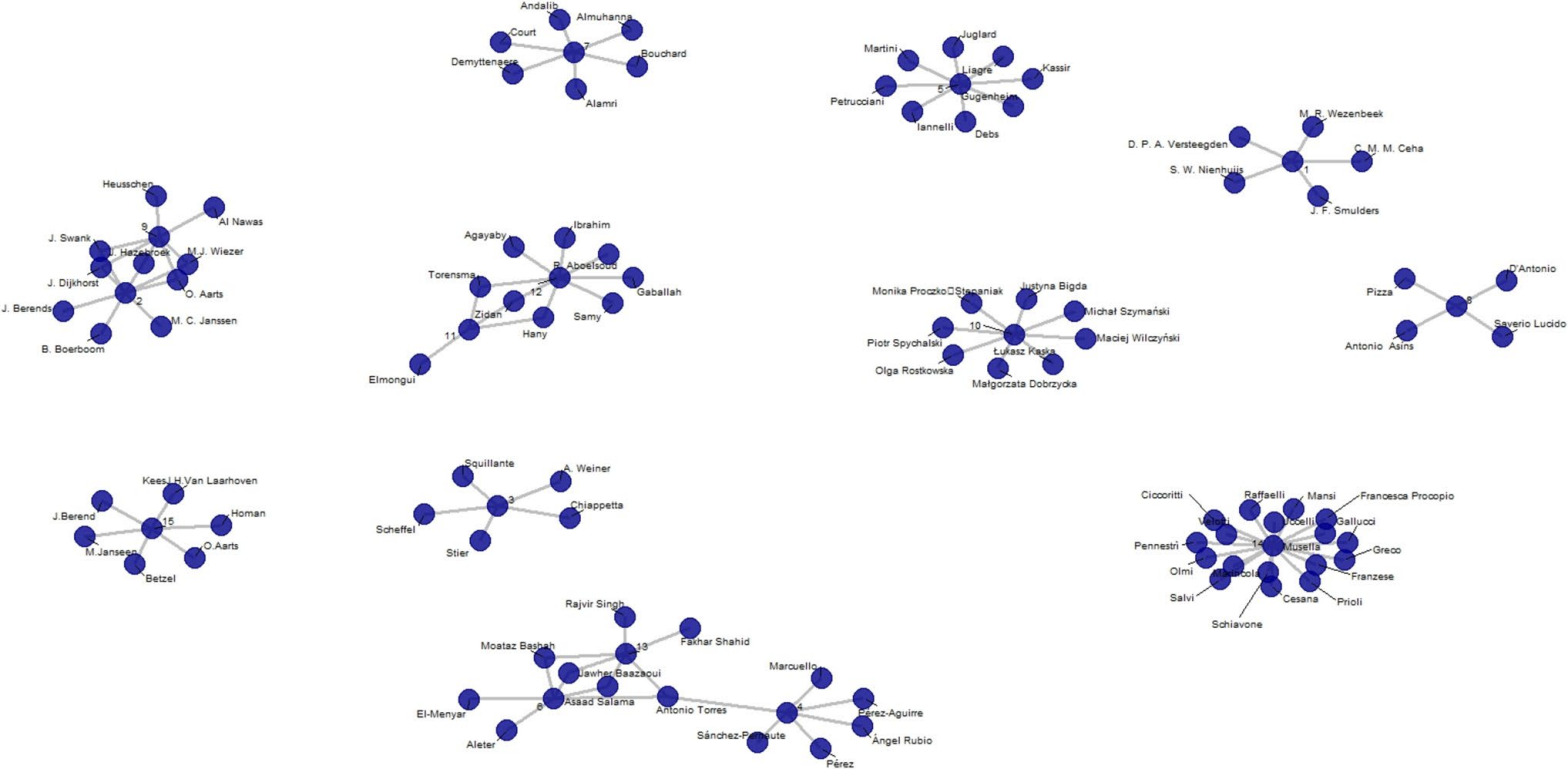
Author co-author network graph for the included studies

Fifteen studies reporting nutritional outcomes for revisional bariatric surgery after failed LSG were included and presented chronologically (Table 1). A co-author network graph was generated to identify potential connections or duplications among the included studies (Fig. 1).

**Table 1:** Summary table of the included studies

ID	Author et al. year	Design	Sample size	Follow up (months)	Outcomes	Revisional surgery	Indications for revisional surgery	Sample size for each revisional surgery	Mean age	Mean BMI	Female gender N (%)
1	Homan et al. 2014 [16]	Retrospective cohort	43	Median = 42	Vit D, B12, Albumin, Iron, Ferritin, Anemia, Calcium, Zn	Biliopancreatic Diversion with Duodenal Switch (BPD-DS)	- Weight regain, insufficient weight loss - Gastroeophageal reflux, dysphagia,	25	40.3	60	14 (56%)
				Median = 29		RYGB		18	48.5	49	8 (44%)
2	Ceha et al. 2018 [17]	Retrospective cohort	64	6	Vit. D, Vit. B12	SADI-S	-Insufficient weight loss -Weight regain after primary SG	32	46.9	57.5	26 (81.3%)
						RYGB		32	48.2	53.7	26 (81.3%)
3	Dijkhorst et al. 2018 [18]	Retrospective cohort	140	24	Vit. D, Vit. B12, Calcium, Albumin, Parathyroid, Folate, Ferritin, Anemia, Vit. B1, Vit. B2	SADI-S	-Weight regain or insufficient weight loss -Functional problem with the SG (e.g. stenosis, reflux, or fistula) -combination of both	66	43.3	45.6	55 (83.3%)
						RYGB		74	45.1	39.3	59 (79.7%)
4	Chiappetta et al. 2019 [19]	Prospective cohort	55	12	Signs of nutritional deficiencies (fatigue, hair loss, glossitis, neuropathic pain, dystrophic nails…)	OAGB	-Weight regain (> 15% of their 1-year postoperative weight) -Insufficient weight loss (EWL < 50%) -intractable GERD (esophagitis ≥ grade B according to the Los Angeles Classification and a GERD-HRQL score ≥ 12 in PPI)	34	46.76	45.7	23 (67.6%)
						RYGB		21	46.14	36.6	19 (90%)
5	Sanchez-Pernaute et al. 2020 [20]	Retrospective cohort	51	60	Hemoglobin, Hematocrit, Iron, Calcium, parathyroid, Vit. D, Copper, Selenium, Zinc, Proteins, Albumin, ALT, AST, GGT	SADI-S	-Weight regain -Insufficient weight loss -Absence of metabolic improvement	51	42	52	35 (68.6%)
6	Debs et al. 2020 [21]	Retrospective cohort	77	55	Albumin, Pre-albumin, Vit. A, Vit. B12, Hemoglobin, Ferritin, Vit. D, Calcium	OAGB	-Weight loss failure defined as %EWL < 50% at 18 m. after surgery	77	45.3	42.03	63 (81.8%)
7	Bashah et al. 2020 [22]	Retrospective cohort	91	12	HbA1C, Total serum protein, Hemoglobin, Albumin, Zinc, Vit. B12, INR, Vit. D, Triglycerides, Cholesterol, Iron, HDL, LDL	SADI-S	-Weight regain post LSG	42	38	43.7	30 (71.4%)
						OAGB		49	37.83	43.6	42 (85.7%)
8	Andalib et al. 2021 [23]	Retrospective cohort	94	18	Protein-caloric malnutrition	single-anastomosis duodenal switch (SADS)	-Primary inadequate weight loss (< 50% EWL) -Weight regain (≥ 20% regain of weight lost) -Persistent obesity-related comorbidity	7	Median = 35	Median = 46.6	5 (71.4%)
						RYGB		41	Median = 51	Median = 40.6	35 (85.4%)
						Re-sleeve		13	Median = 53	Median = 39.5	12 (92.3%)
						Duodenal switch		33	Median = 44	Median = 46.8	25 (75.8%)
9	Pizza et al. 2021 [24]	Retrospective cohort	59	Mean = 34.32	Vit. D, Vit. B12, Albumin, Sreum Iron	OAGB	A failure of SG defined as: -Insufficient weight loss -Weight regain	59	43.08	43	35 (59.3%)
10	Dijkhorst et al. 2021 [25]	Retrospective cohort	141	Mean = 55.2	Calcium, Sreum Iron, Vit. D, Albumin, Vit. B12, Total serum protein, Parathyroid, B1, B6, Mg, Zn, Ferritin	SADI-S	-Insufficient weight loss -functional problems related to the SG -combination of both	63	43.6	44.9	53 (84.1%)
				Mean = 93.6		RYGB		78	46	39.1	61 (78.2%)
11	Wilczyński et al. 2022 [26]	Case control	80	60	Anemia, hemoglobin, iron, Vit B12, Vit D, Albumin	OAGB	-Failure of LSG treatment defined as unsatisfactory weight loss (defined when the BMI ≥ 35 kg/m2 and EWL < 50%) -Resistance to the medical treatment for symptomatic GERD after endoscopic evaluation	47	45.02	40.44	34 (72.3%)
						RYGB		33	41.24	38.70	27 (81.8%)
12	Hany et al. 2022 [27]	Randomized clinical trial	160	24	Hemoglobin, Hemoglobin A1c, Albumin, Calcium, Vit. B12, Vit. D, Parathyroid, Lipid profile, Ferritin	OAGB	-Weight regain defined as an increase in BMI after bariatric surgery to ≥ 35	80	42.6	45.1	69 (86.3%)
						RYGB		80	43.4	44.9	69 (86.3%)
13	Hany et al. 2023 [28]	Randomized clinical trial	50	24	Nutritional deficiencies after two years: Ferritin, Calcium, Vit. D, Vit. B12, Albumin, Hemoglobin	Banded LSG	-Weight regain exceeding their nadir, reported as a BMI ≥ 35	25	40.7	47.7	21 (84.0%)
						Non-banded LSG		25	39.7	46.9	17 (68.0%)
14	Salama et al. 2023 [29]	Retrospective cohort	91	36	Total serum protein, Vit. D, Vit. B12, Lipid profile, Serum Iron, Hemoglobin, INR, Zinc	OAGB	-Weight regain	49	37.83	43	42 (85.7%)
				60		SADI-S		42	38	45.9	30 (71.4%)
15	Gallucci et al. 2024 [30]	Prospective cohort	84	24	Malnutrition	OAGB	-Recurrence of weight: defined by a post-operative weight increase ≥ 50% of the lowest weight achieved after the initial surgery -Suboptimal clinical response: defined as %EWL < 50% at the post-operative nadir weight	42	41.2	43.8	27 (64.3%)
						SADI-S		42	42.9	44.8	33 (78.6%)

### Co-Author Network Analysis

The co-author network revealed multiple links between several studies. Upon meticulous review, Sanchez-Pernaute et al. (2020), Basha et al. (2020), and Salama et al. (2023) were found to share overlapping authorship [20, 22, 28]. Hany et al. (2022) and Hany et al. (2023) were determined to be completely different studies focusing on distinct revisional bariatric procedures [27, 29]. Dijkhorst et al. (2018) and Dijkhorst et al. (2020) involved the same cohort with varying follow-up periods [18, 25]. Similarly, Basha et al. (2020) and Salama et al. (2023) analyzed the same cohort with different follow-up durations [22, 28], raising concerns about duplication of baseline and outcome data. To address this, the decision was made to include only the most recent study in the analysis to ensure the most comprehensive and updated data, while avoiding redundancy and duplicated cohorts.

### Baseline Characteristics

After excluding similar cohorts, the study included a total of 1,049 patients who underwent revisional bariatric surgery, of whom 801 were female (76%). The mean age across all patients was 44.2 years, with a mean BMI of 45.97 kg/m2. Follow-up durations ranged from 6 to 96 months. All included studies reported weight regain or failure to lose weight as the primary indication for revision surgery (Table 1).

When stratified by type of revisional procedure, 388 patients underwent OAGB, with a mean age of 43.11 years, a BMI of 43.16 kg/m2, and females comprised 78% of this group. Patients revised to RYGB totaled 303, with a mean age of 46.57 years, a BMI of 43.23 kg/m2, and 80.1% were female. The SADI-S group included 237 patients, with a younger mean age of 42.5 years, a higher BMI of 49.02 kg/m2, and females represented 69% of this group.

### Quality Assessment

In this systematic review, to ensure the accuracy of the assessment of the validity of the included studies, we applied the Cochrane risk of bias 2.0 (RoB 2) tool for randomized controlled trials (RCTs) [30]. These tools evaluate the two included RCTs based on these five domains: (randomization process, deviations from intended interventions, missing outcome data, measurement of the outcome, and selection of the reported result), thus increasing the accuracy and reliability of our results (supplementary appendix).

For the 12 included cohort studies and one case–control study, we used the Newcastle–Ottawa Scale tool (NOS) [14], which assessed them according to those categories: selection, which could be awarded a maximum of 4 stars, comparability (maximum 2 stars), and outcome (maximum 3 stars). The overall results vary between moderate and low risk of bias (supplementary appendix). Limitations across almost all studies include a lack of clarity on follow-up duration and potential outcome assessment bias due to the observational nature of the data.

### Nutritional Outcomes

#### Vitamin D

**Fig. 2:**
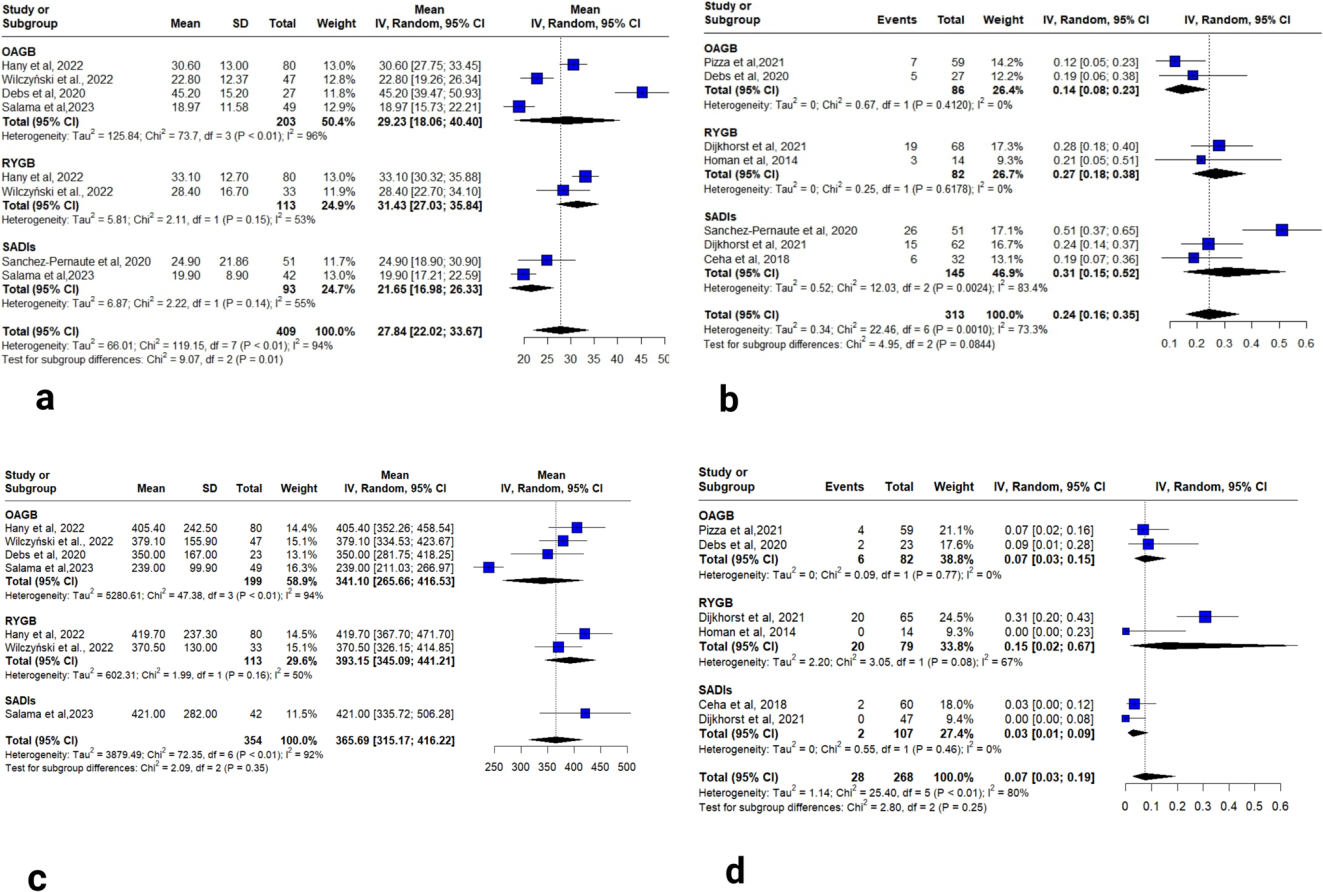
**a** forest plot of vitamin D level, **b** forest plot of vitamin D deficiency, **c** forest plot of vitamin B12 level, **d** forest plot of vitamin B12 deficiency

Of the 15 included studies, 12 examined Vitamin D outcomes, five reported continuous data and six provided categorical data on deficiency rates. In studies assessing mean Vitamin D levels [20, 21, 26, 27, 28], OAGB group yielded a pooled mean of 29.23 ng/mL (95% CI: 18.86–40.40; I2 = 96%), which dropped after sensitivity analyses with pooled mean = 20.82 (95% CI: 17.07–24.57; I^2^ = 59.1) In comparison, the RYGB group yielded a pooled mean of 31.43 ng/mL (95% CI: 27.03–35.84; I2 = 53%). For the SADI-S group, the studies reported a pooled mean of 21.65 ng/mL (95% CI: 16.98–26.33; I2 = 55%) (Fig. 2a). % A statistically significant difference was found between the RYGB and SADI-S groups (*p* < 0.01).

Regarding the studies reported, the vitamin D deficiency categorically [16–18, 20, 21, 24, 25]. For the OAGB group, a deficiency rate of 0.14 (95% CI: 0.08–0.23; I2 = 0%) was reported. For RYGB, a deficiency rate, reported by two studies, was 0.27 (95% CI: 0.18–0.38; I2 = 0%). In the SADI-S group, a pooled rate of 0.31 (95% CI: 0.15–0.52; I2 = 83%) was reported (Fig. 2b). A sensitivity analysis was done, giving a pooled rate of 0.22, by excluding Sánchez-Pernaute et al. which dropped the I^2^ to 0% [20]. No statistically significant difference was found between groups (*p* = 0.08).

#### Vitamin B12

Of the 15 included studies, 12 examined Vitamin B12 outcomes, four reported continuous data, and five provided categorical data on deficiency rates. In studies assessing mean Vitamin B12 levels [21, 22, 26, 27, 28]. For the OAGB group, the presented pooled mean was 341.10 pg/mL (95% CI: 265.66–416.53; I2 = 94%), with heterogeneity eliminated (I2 = 0%) upon exclusion of Salama et al. (2023), with pooled mean 381.10 pg/mL (95% CI: 351.42–412.50; I2 = 94%) [28]. In the RYGB group, the reported pooled mean was 393.15 pg/mL (95% CI: 345.09–441.21; I2 = 50%). One study reported a mean of 421 pg/mL (95% CI: 335.72–506.28) for the SADI-S group [28] (Fig. 2c). There were no significant differences between the three groups (*p* = 0.35), even after the sensitivity analysis was done (*p* = 0.68).

Regarding the studies reported, the vitamin B12 deficiency categorically [16–18, 21, 24, 25], a deficiency rate of 0.07 (95% CI: 0.03–0.15; I2 = 0%) in the OAGB group. In contrast, the RYGB group reported a rate of 0.15 (95% CI: 0.02–0.67; I2 = 67%). For SADI-S, a pooled rate of 0.03 (95% CI: 0.01–0.0.9; I2 = 0%) (Fig. 2d). There were no significant differences between the three groups (*p* = 0.25).

#### Albumin

**Fig. 3:**
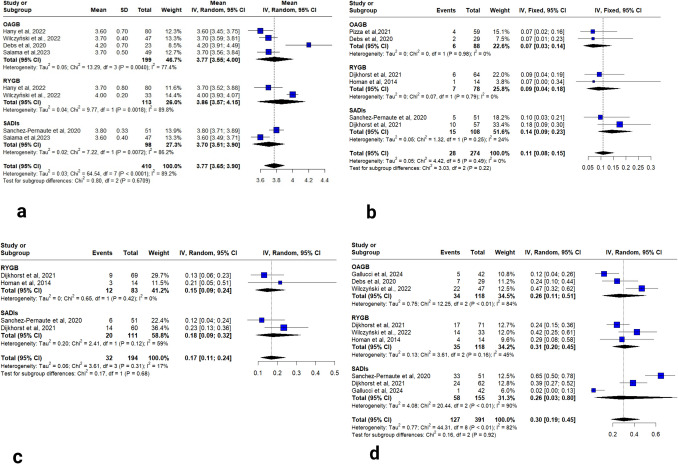
**a** forest plot of albumin level, **b** forest plot of albumin deficiency, **c** forest plot of calcium deficiency, **d** forest plot of anemia incidence

Out of the 15 included studies, 5 evaluated albumin as a continuous outcome [21, 22, 24, 26, 27, 28]. For OAGB, the reported pooled mean albumin level was 3.77 g/dL (95% CI: 3.55–4.00; I^2^ = 77.4%), which dropped by excluding Debs et al. (I^2^ = 0) [21]. For RYGB, the reported pooled mean was 3.86 g/dL (95% CI: 3.57–4.15; I^2^ = 89.8%). In the SADI-S group, the reported pooled mean was 3.7 g/dL (95% CI: 3.51–3.90; I^2^ = 86.2%) (Fig. 3a). No statistically significant difference in continuous albumin levels was observed between the three groups (*p* = 0.67).

In the analysis of categorical data (albumin deficiency), Five studies were included [16, 18, 20, 21, 24, 25]. For OAGB, the reported pooled deficiency rate was 0.07 (95% CI: 0.03–0.14; I^2^ = 0). For RYGB, the reported rate was 0.09 (95% CI: 0.04–0.18; I^2^ = 0). In the SADI-S group, the reported pooled rate was 0.14 (95% CI: 0.09–0.23; I^2^ = 24) (Fig. 3b). No statistically significant difference was found between groups (*p* = 0.23).

#### Calcium

Three studies reported calcium deficiency as categorical data [16, 18, 20, 25]. Two studies reported a pooled deficiency rate of 0.15 (95% CI: 0.09–0.24; I^2^ = 0) in RYGB [16, 25]. In addition, the pooled deficiency rate was 0.18 (95% CI: 0.09–0.32; I2 = 0%) in the SADI-S group (Fig. 3c). There was no significant difference between RYGB and SADI-S (*p* = 0.52).

#### Anemia

Out of the 15 included studies, 6 evaluated anemia incidence [16, 18, 20, 25, 26, 31]. The OAGB group yielded a pooled rate of anemia of 0.26 (95% CI: 0.11–0.51; I^2^ = 84), which dropped by excluding Wilczyński et al. (2022) with a pooled rate of 0.17 (95% CI: 0.08–0.33; I^2^ = 43) [26]. The RYGB group yielded a pooled rate of anemia of 0.31 (95% CI: 0.20–0.45; I^2^ = 45). In addition, the SADI-S group yielded a pooled rate of 0.26 (95% CI: 0.03–0.8; I^2^ = 90), and showed persistent heterogeneity with sensitivity analysis (Fig. 3d). No significance was detected between different procedure groups (*P* = 0.9).

#### Iron

**Fig. 4:**
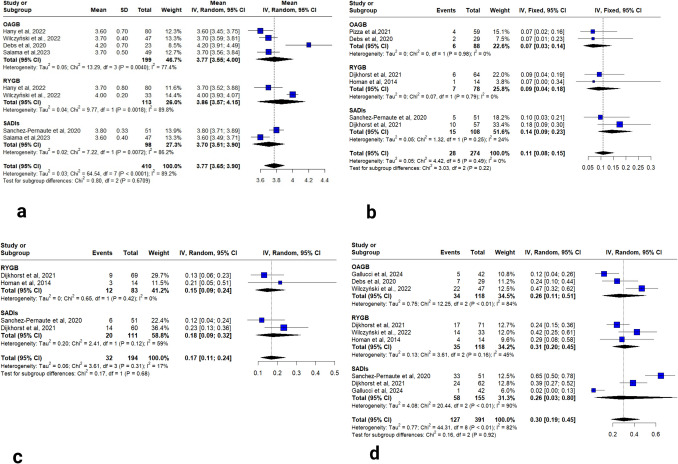
**a** forest plot of iron level, **b** forest plot of iron deficiency (**c**) forest plot of (**d**) forest plot of

Out of the 15 included studies, 3 evaluated iron levels [20, 22, 26, 28]. For OAGB, the reported pooled mean was 11.89 μmol/L (95% CI: 10.53–13.24; I^2^ = 0). For RYGB, the reported mean was 12.15 μmol/L (95% CI: 10.34–14.62). In the SADI-S group, the reported pooled mean was 12.22 μmol/L (95% CI: 10.34–14.09; I^2^ = 38.4) (Fig. 4a). No statistically significant difference was observed between the OAGB, RYGB, and SADI-S groups in terms of continuous iron levels (*p* = 0.95).

In the analysis of categorical data (iron deficiency) [20, 24, 25]. For OAGB, the reported deficiency rate was 0.12 (95% CI: 0.05–0.23). For RYGB, the reported rate was 0.17 (95% CI: 0.07–0.34). In the SADI-S group, the reported a pooled rate was 0.31 (95% CI: 0.22–0.42; I^2^ = 0) (Fig. 4b). A statistically significant difference in iron deficiency rates was found between the three groups (*p* = 0.025), with a significant difference between OAGB and SADI-S (*p* = 0.0106).

#### Ferritin

Three studies reported ferritin deficiency as categorical data [20, 21, 45]. Two studies reported a pooled deficiency rate of 0.25 (95% CI: 0.17–0.36; I^2^ = 0) in RYGB. In addition, the deficiency rate was 0.12 (95% CI: 0.05–0.23) in the OAGB group, and 0.33 (95% CI: 0.22–0.46) In the SADI-S group (Fig. 4c).

#### Zinc

Three studies evaluated Zinc deficiency as categorical data [16, 21, 45]. The pooled deficiency rate was 0.09 (95% CI: 0.02–0.37; I2 = 0%) in the RYGB group and 0.55 (95% CI: 0.44–0.66; I2 = 0%) in the SADI-S group (Fig. 4d). The difference between RYGB and SADI-S was statistically significant (*p* < 0.01).

#### Other Bariatric Revisional Surgeries

Other revisional bariatric procedures have been less extensively investigated in the literature. In a study by Hany et al. [29], banded versus non-banded LSG showed no significant differences in nutritional deficiencies, including calcium, ferritin, vitamin D, vitamin B12, albumin, and hemoglobin, after two years of follow-up (*p* ≥ 0.110). Similarly, Andalib et al. reported that while biliopancreatic diversion with duodenal switch BPD-DS was linked to a higher, though statistically insignificant, rate of protein-calorie malnutrition compared to re-sleeve gastrectomy, RYGB, and single-anastomosis duodenal switch (SADS) (P = 0.381), re-sleeve had similar malnutrition rates to RYGB and SADS [23]. Homan et al. compared nutritional outcomes between BPD-DS and RYGB. Anemia occurred in 45% of BPD-DS patients versus 29% after RYGB, calcium deficiency in 14% versus 21%, and zinc deficiency in 27% versus none. Vitamin D deficiency was present in 55% of BPD-DS patients compared with 21% after RYGB, while no patients in either group developed vitamin B12 deficiency [16].

## Discussion

LSG is among the most commonly performed metabolic surgeries. As such, postoperative nutritional challenges are anticipated, necessitating long-term clinical follow-up. Studies report that approximately 15–20% of patients undergoing LSG require revisional surgery due to various factors, including technical complications, persistent nutritional deficiencies, or inadequate weight loss outcomes [7, 8]. Revisional bariatric surgeries have been steadily increasing since 2011, particularly in specialized centers in the USA and subsequently around the world [32]. With the increasing prevalence of bariatric surgery, several secondary procedures have been developed to enhance outcomes and address limitations of earlier techniques. Among these, OAGB and SADI-S have gained popularity. These procedures are favored for their technical simplicity, shorter operative times, preservation of a larger gastric remnant, and their ability to achieve effective weight loss [9].

In our systematic review, we included 15 studies [16–27, 28, 29, 30] which state that OAGB already represents the single largest revision category (388/1,049 patients, 36.9%), superior of RYGB (303/1,049, 28.9%) and SADI-S (237/1,049, 22.6%), indicating a shift towards single-anastomosis approaches in the included studies. However, it is important to note that this distribution reflects only the subset of literature focused on nutritional outcomes after revision and should not be interpreted as a representation of the global tendency in all revisional bariatric practices.

OAGB, the most represented revisional surgery in our systematic review, involves the creation of a long tubular gastric pouch anastomosed to a jejunal loop approximately 150–200 cm distal to the ligament of Treitz [33]. This configuration partially bypasses the duodenum while preserving some of its absorptive function. However, deficiencies in vitamin B12 and iron remain concerns. When the biliopancreatic limb exceeds 200 cm, there is an increased risk of protein-calorie malnutrition [34].

RYGB entails the formation of a small gastric pouch, with a biliopancreatic limb and an alimentary limb. This bypasses the duodenum and proximal jejunum, which are essential for the absorption of calcium and vitamin B12. In cases where the biliopancreatic limb is longer, fat-soluble vitamin deficiencies may also occur. Patients typically require lifelong multivitamin supplementation, including calcium, vitamin D, and B12 [35, 36].

SADI-S, the third most represented technique, combines a vertical sleeve gastrectomy with a duodeno-ileal anastomosis approximately 250–300 cm proximal to the ileocecal valve. This significantly shortens the common channel, where most micronutrient absorption occurs. As a result, SADI-S is associated with the highest incidence of fat-soluble vitamins (A, D, E, K), iron, and protein deficiencies. Management involves high-dose fat-soluble vitamin supplementation, 60–80 g of protein daily, and 65–100 mg of iron, vitamin B12, and trace elements [37]. Most of the included studies in our review reported the use of supplementation regimens to address these nutritional deficiencies [17, 18, 20, 22–29]. However, the occurrence of multiple deficiencies may still arise, either due to patient non-compliance or as an inherent consequence of the surgical technique.

In accordance, Abedalqader et al. demonstrated that OAGB is increasingly adopted in high-risk patients with lower tolerance for severe malabsorption and can be safely used in elderly and vegan individuals, while SADI-S may be reserved for super-obese patients who can adhere to intensive nutritional follow-up [38]. Different metabolic and bariatric surgeries can alter gastrointestinal anatomy in distinct ways, which impacts how nutrients are absorbed. These surgical variations lead to specific nutritional deficiencies for according to each technique and influence the amount of supplementation required postoperatively.

Accordingly, the included studies reported that all patients were prescribed a multivitamin protocol [17, 18, 20, 22–29], except in the studies by Chiappetta et al. Debs et al. and Gallucci et al. [19, 21, 31], highlighting the importance of standardized supplementation protocols and rigorous methodology in revisional bariatric surgery. These findings align with the nutritional outcomes and deficiencies highlighted in our systematic review, emphasizing the impact of surgical technique, the necessity of multivitamin prescription, and the importance of assessing patient compliance.

The American Society of Metabolic and Bariatric Surgery (ASMBS) recommends that all patients preparing for MBS undergo a thorough preoperative evaluation of their nutritional health, which includes checking micronutrient levels such as thiamine, folic acid, vitamin B12, vitamins A, E, D, and K, along with calcium, iron, zinc, and copper. This evaluation not only detects and allows for correction of existing deficiencies but also provides a baseline for postoperative comparison [39]. Moreover, the presence of preoperative micronutrient deficiencies has been shown to predict their persistence following surgery [40].

To ensure reliable results, we examined the authorship of included studies to identify potential data overlaps. For instance, Basha et al. and Salama et al. shared six authors and analyzed the same SADI-S versus OAGB cohorts at different follow-up intervals [22, 28], while Dijkhorst et al. (2018) and Dijkhorst et al. (2021) used the same Dutch multicenter registry with extended follow-up [18, 25]. To avoid duplication and skewed effect estimates, we included only the most recent report from each pair. Although this reduced the sample size, it enhanced the validity of our findings.This methodological refinement underscores the importance of meticulously reviewing included studies in systematic reviews. We also propose using co-author network graphs as a helpful tool for identifying potential overlaps in study populations [41]

Vitamin D is among the micronutrient deficiencies that commonly arise after MBS, with significant implication on bone health and the potential to cause long-term complications. Vitamin D depends on bile salts for its passive absorption in the jejunum and ileum. Since MBS result in anatomical alterations of the GI tract, they disrupt efficient absorption of vitamin D [42]. In our analysis, the vitamin D deficiency rate was numerically higher in the RYGB group (27%) compared to the OAGB group (14%), although this difference did not reach statistical significance (*p* = 0.08). The higher rate observed after RYGB may be explained by its anatomical configuration, as bypassing the duodenum and proximal jejunum limits adequate mixing of vitamin D with bile salts, thereby hindering its absorption [36].

Vitamin B12 deficiency commonly arises after MBS due to reduced stomach volume thereby, reduced gastric acid and intrinsic factor secretion, which are both essential for B12 absorption [43]. Pooled analysis of deficiency rates showed lower rates in the SADI-S (3%) and OAGB (7%) groups compared to RYGB (15%). However, these differences were not statistically significant (*p* = 0.25). This is attributed to the complete bypass of stomach and proximal small bowel in the RYGB procedure, where the intrinsic factor is produced and vitamin B12 is absorbed. However, OAGB and SADI-S may preserve larger portion of the stomach so some intrinsic factor is still produced [17].

Across the studies included in this review, no statistically significant differences in mean serum iron levels were identified between the various bariatric revision procedures. However, categorical assessments of deficiency revealed a different pattern. Patients undergoing SADI-S exhibited higher rates of iron deficiency compared to those who underwent OAGB. (*p* = 0.0106). OAGB was more frequently associated with the lowest rates of iron deficiency (*p* = 0.025), suggesting it may have a comparatively lesser impact on iron stores or absorption.

Calcium deficiency remains a clinically relevant postoperative issue following both RYGB and SADI-S procedures. Although the studies reported small differences in the rate of calcium deficiency, none reached statistical significance [16, 20, 25]. These results highlight the importance of long-term monitoring of calcium levels and bone mineral density, and the necessity of patient adherence to supplementation protocols.

No significant differences in mean serum albumin levels were noted between revision procedures. However, a slightly increased incidence of hypoalbuminemia was observed in patients who underwent the SADI-S procedure [16, 20, 21, 24, 25]. While this did not reach statistical significance, it may reflect a tendency toward greater protein hypoabsorption.

There were no significant differences in anemia rates detected between the different revisional procedures (*P* = 0.92). The OAGB group demonstrated the lowest anemia incidence of 17% (95% CI: 0.08–0.33; I2 = 43). These findings suggest that anemia remains a shared concern across revision types, potentially influenced by differing follow-up durations, supplementation adherence, or unmeasured baseline patient characteristics.

Any revisional bariatric surgery involving a malabsorptive component may result in malnutrition with varying degrees, partly due to the altered anatomy and partly due to poor compliance of patients to proper diet and multivitamin supplements [44]. Therefore, regular monitoring of micronutrients levels is essential in post-operative management to detect any early deficiency and allow for timely intervention before long-term complications occur.

Plath et al. reported 48 cases of RYGB reversal, half of which had already undergone at least one prior bariatric revision. In 12.5% of these cases, malabsorption was the indication for reversal [45]. Similarly, Kermansaravi et al. demonstrated that the major signs and symptoms of protein-energy malnutrition were among the leading causes for OAGB reversal [46], underscoring the importance of consistent nutritional monitoring.

While the first year following bariatric surgery is critical for identifying early micronutrient deficiencies, evidence suggests that deficiencies may develop beyond this period [47], underscoring the necessity of implementing a structured, long-term monitoring strategy. The ASMBS recommends monitoring up to ten micronutrients every three months during the first postoperative year, followed by assessment every six months in the second year, and annually thereafter, irrespective of the type of procedure performed [48]. While, the British Obesity and Metabolic Surgery Society (BOMSS) recommends a more individualized approach, focusing on five key nutrients at three intervals within the first year while reserving additional tests for specific clinical indications or annual follow-ups [49].

While efforts were made to assess the methodological quality of the included studies, a formal evaluation of publication bias was not feasible due to the limited number of studies, which restricted the use of visualization tools like funnel plots or other standard techniques. Moderate heterogeneity was observed in several reported outcomes. This variability may be attributed to differences in study design, such as the retrospective nature of many studies, inconsistent definitions of nutritional deficiencies, variation in surgical techniques, and differing baseline patient characteristics.

Additionally, the duration of follow-up varied significantly among the included studies, which likely influenced the detection of nutritional complications. Short-term studies may underestimate late-onset issues such as progressive micronutrient depletion or chronic anemia. In contrast, cohorts with longer follow-up periods revealed deficiencies that were not evident in the early postoperative phase, underscoring the importance of long-term nutritional monitoring in this population [44].

The single-arm analyses presented are not definitive. A significant limitation of the current evidence is the lack of sufficient data to compare post-revision nutritional outcomes directly against pre-revision baseline status. This gap makes it difficult to isolate the specific impact of the revisional surgery itself from the pre-existing deficiencies caused by the initial sleeve gastrectomy. Therefore, large-scale clinical trials comparing different bariatric revision procedures from a nutritional perspective are urgently needed. These must incorporate rigorous follow-up durations and critically standardized, well-documented pre-revision baseline measurements to establish a clear starting point and ensure valid and meaningful comparisons.

In our systematic review, we aim to highlight the importance of nutritional outcomes in revisional bariatric surgery, in order to validate the overall benefits for bariatric surgery patients, not only in terms of weight loss but also in minimizing the risk of nutritional deficiencies. Given the varying incidence of these deficiencies across different revisional bariatric procedures, this concern warrants greater attention to ensure comprehensive patient care.

## Conclusion

This systematic review highlights the importance of monitoring nutritional outcomes in revisional bariatric surgery after LSG. While these procedures can be effective, those with malabsorptive effects carry a higher risk of micronutrient and protein deficiencies. Despite the increasing number of revision surgeries, many studies continue to underreport or inadequately monitor nutritional status, underscoring the need for standardized follow-up protocols.

## Supplementary Information

Below is the link to the electronic supplementary material.

Supplementary file 1 (DOCX 1.67 MB)

## Data Availability

No datasets were generated or analysed during the current study.

## Abbreviations

*ASMB*: American Society of Metabolic and Bariatric Surgery
*BOMSS*: British Obesity and Metabolic Surgery Society
*BPD-DS*: biliopancreatic diversion with duodenal switch
*GERD*: gastroesophageal reflux disease
*LSG*: laparoscopic sleeve gastrectomy
*MBS*: metabolic and bariatric surgeries
*OAG*: Bone anastomosis gastric bypass
*PRISMA*: Preferred Reporting Items for Systematic Reviews and Meta-Analyses
*RYG*: Broux-en-Y gastric bypass
*SADI-S*: single anastomosis duodeno-ileal bypass with sleeve
*SADS*: single-anastomosis duodenal switch
