# Supplementary material for: The Nutritional Challenges Following Revisional Bariatric Surgery After Sleeve Gastrectomy: A Systematic Review and Meta Analysis
Source: Obes Surg. 2025 Nov 5;35(12):5515–30. doi: 10.1007/s11695-025-08325-2 (PMC12722500; doi:10.1007/s11695-025-08325-2)

**Supplementary** **appendix**

This supplementary material for the article entitled: The Nutritional Concerns After Revisional Bariatric Surgeries due to Failed Sleeve Gastrectomy: Systematic Review and Meta-Analysis

[Figure S1 PRISMA flow diagram of included studies 2](#_Toc203035541)

[Figure S2 RoB assessment using the risk-of-bias 2 assessment tool for included randomized clinical trials 3](#_Toc203035542)

[Table S1 Summary of RoB assessment using the Newcastle-Ottawa Quality Assessment Scale for cohort studies 4](#_Toc203035543)

[Table S2 Summary of RoB assessment using the Newcastle-Ottawa Quality Assessment Scale for the included case-control study 5](#_Toc203035544)

[Figure S3 Mean Vitamin D deficiency Sensitivity analysis 6](#_Toc203035545)

[Figure S4 Subgroup of Mean Vitamin D deficiency for RYGB and SADI-S 6](#_Toc203035546)

[Figure S5 Subgroup of Mean Vitamin D deficiency for OAGB and RYGB 7](#_Toc203035547)

[Figure S6 Subgroup of Mean Vitamin D deficiency for OAGB and SADI-S 7](#_Toc203035548)

[Figure S7 Vitamin D deficiency Sensitivity Analysis 8](#_Toc203035549)

[Figure S8 Mean Albumin Sensitivity analysis 9](#_Toc203035557)

[Figure S9 Anemia Sensitivity analysis 10](#_Toc203035558)

Figure S10 subgroup of Iron deficiency for OAGB and SADI-S……………………………………………………………..10

Figure S11 analysis of ferritin deficiency ................................................................................................11

Figure S12 Zinc deficiency analysis.........................................................................................................11

## Figure S1 PRISMA flow diagram of included studies

Records identified from:

Databases (n =653)

PubMed (n = 200)

Scopus (n = 284)

Web of Science (n = 151)

Cochrane (n = 18)

**Identification**

**Included**

Records screened primarily

(n =479)

Records excluded

(n =436)

Review article (n = 28)

Letters and editorials (n=19)

Animal studies (n=11)

Reports sought for retrieval

(n =43)

Reports assessed for eligibility

(n =39)

Studies included in review

(n =15)

Studies included from manual search

(n =6)

**Screening**

**Identification of studies via databases and registers**

Records removed *before screening*:

Duplicate records removed (n = 174)

Reports excluded:(n =30)

No nutritional data (n =26)

Endoscopic-only revisions (n =4)

## Figure S2 RoB assessment using the risk-of-bias 2 assessment tool for included randomized clinical trials

**
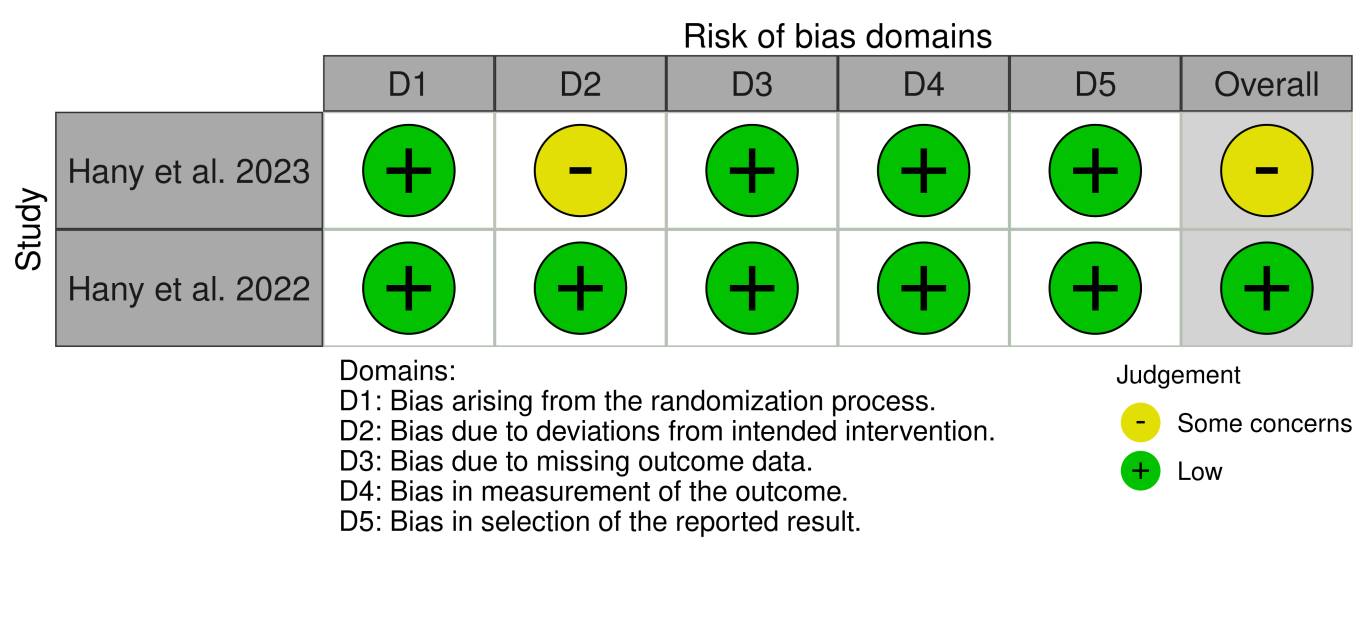
**

## Table S1 Summary of RoB assessment using the Newcastle-Ottawa Quality Assessment Scale for cohort studies

| **Author** | **Year** | **Selection Bias Assessment** | | | | **Comparability** | **Outcome** | | | **Assessor's overall Judgement** |
| --- | --- | --- | --- | --- | --- | --- | --- | --- | --- | --- |
|  |  | Represent-ativeness of the exposed cohort | Selection of the non-exposed cohort | Ascertainm-ent of the exposure (risk factor) | Demonstrat-ion that outcome of interest was not present at the start of the study | Comparability of cohorts on the basis of the design or analysis | Assessment of the outcome | Was follow-up long enough for outcomes to occur? | Adequacy of follow-up of cohorts |  |
| **Homan et al.** | **2014** | ***** | ***** | * | * | * | * | ** | * | Low risk of bias |
| **Ceha et al.** | **2018** | ***** | ***** | * | * | NA | * | * | NA | Low to moderate risk of bias |
| **Dijkhorst et al.** | **2018** | ***** | ***** | * | * | NA | * | * | NA | Low to moderate risk of bias |
| **Chiappetta et al.** | **2019** | ***** | ***** | * | * | NA | * | ***** | ***** | Low risk of bias |
| **Sanchez-Pernaute et al.** | **2020** | ***** | ***** | * | * | NA | * | ***** | ***** | Low risk of bias |
| **Debs et al.** | **2018** | ***** | ***** | * | * | NA | * | ***** | ***** | Low risk of bias |
| **Bashah et al.** | **2020** | * | * | * | * | ** | * | * | NA | Low risk of bias |
| **Andalib et al.** | **2021** | ***** | ***** | * | * | NA | * | ***** | ***** | Low risk of bias |
| **Pizza et al.** | **2021** | * | * | * | * | ** | * | * | * | Low risk of bias |
| **Dijkhorst et al.** | **2021** | ***** | ***** | * | * | NA | * | * | NA | Low to moderate risk of bias |
| **Salama et al.** | **2023** | * | * | * | * | ** | * | * | * | Low risk of bias |
| **Gallucci et al.** | **2024** | * | * | * | * | ** | * | * | * | Low risk of bias |

## Table S2 Summary of RoB assessment using the Newcastle-Ottawa Quality Assessment Scale for the included case-control study

| Domain | Criteria | Stars Awarded |
| --- | --- | --- |
| **Selection** | 1. Is the Case Definition Adequate? | ★ |
| **Selection** | 2. Representativeness of the Cases | ★ |
| **Selection** | 3. Selection of Controls | ★ |
| **Selection** | 4. Definition of Controls | ★ |
| **Comparability** | 1. Comparability of Cases and Controls on the Basis of the Design or Analysis | ★★ |
| **Exposure** | 1. Ascertainment of Exposure | ★ |
| **Exposure** | 2. Non-Response Rate | NA |

## Figure S3 Mean Vitamin D deficiency Sensitivity analysis


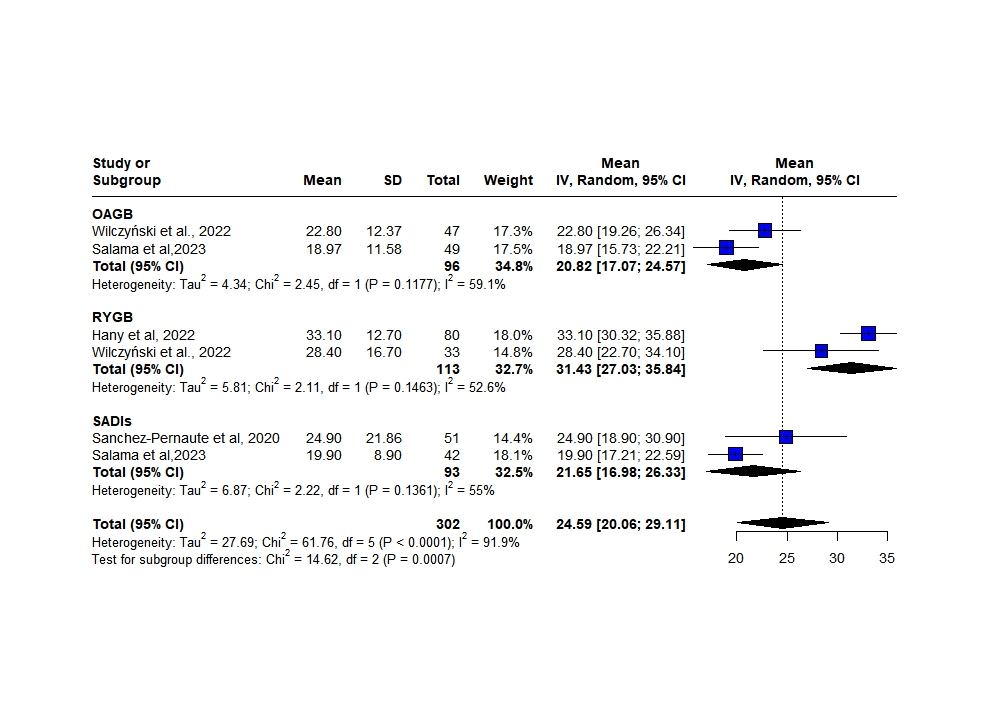


## Figure S4 Subgroup of Mean Vitamin D deficiency for RYGB and SADI-S


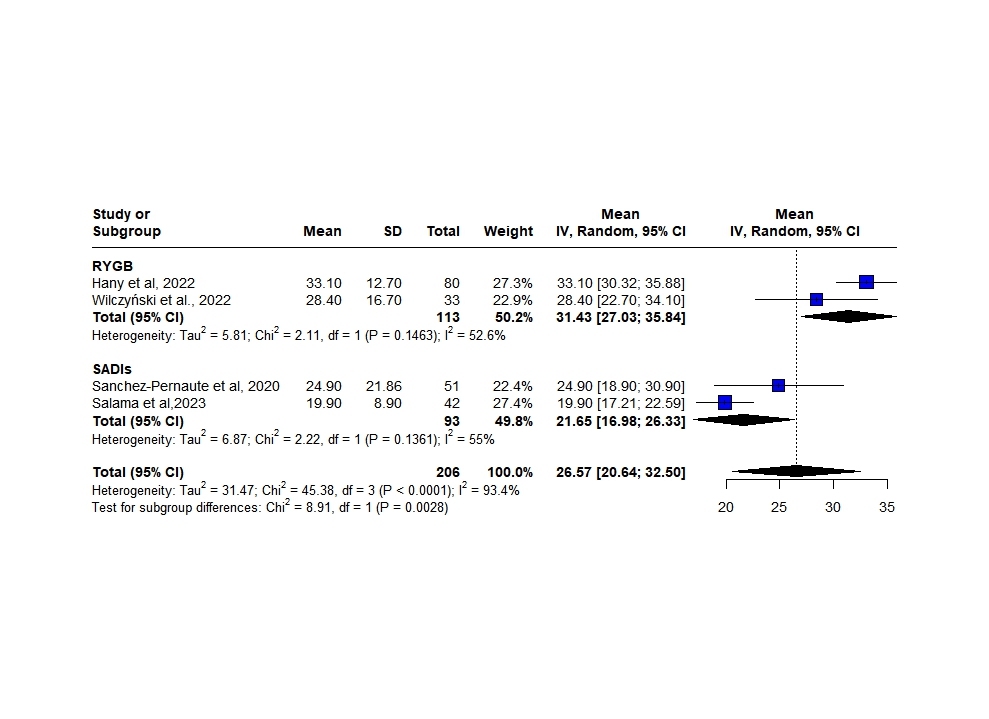


## Figure S5 Subgroup of Mean Vitamin D deficiency for OAGB and RYGB


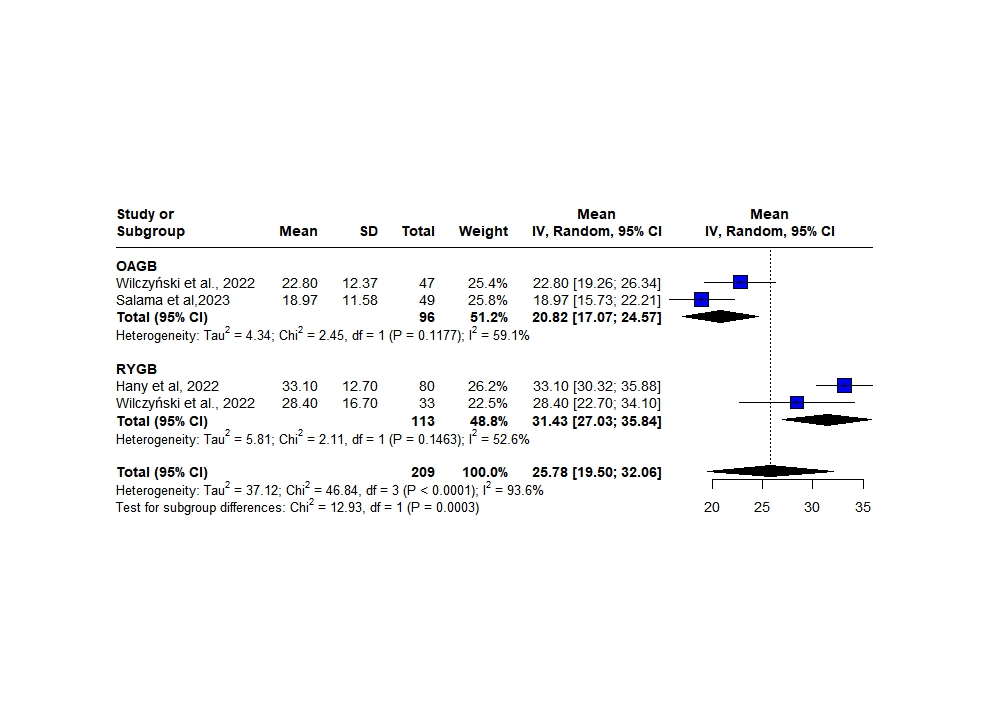


## Figure S6 Subgroup of Mean Vitamin D deficiency for OAGB and SADI-S


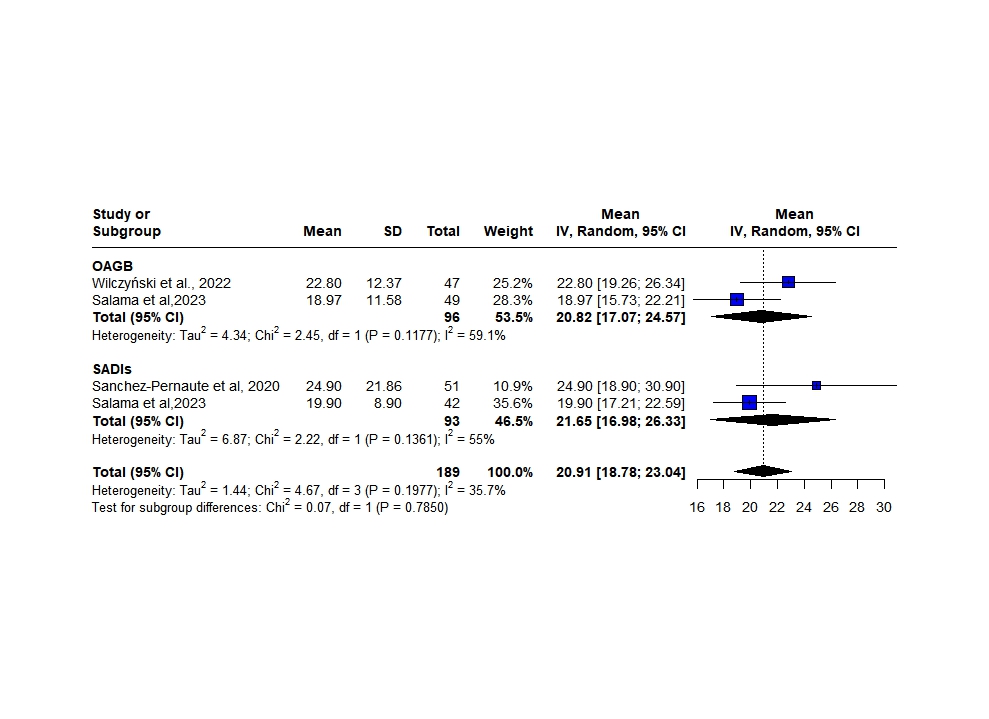


## Figure S7 Vitamin D Deficiency Sensitivity Analysis


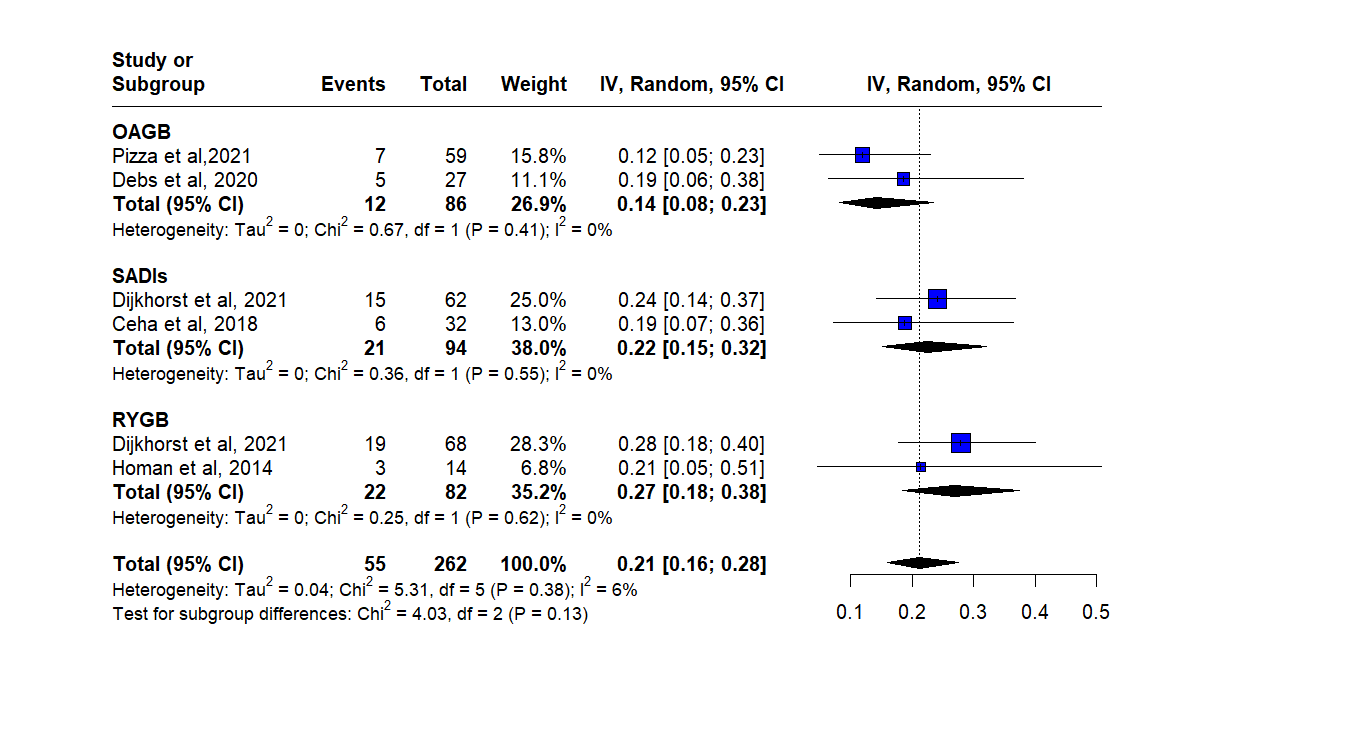


##

## Figure S8 Mean Albumin Sensitivity analysis


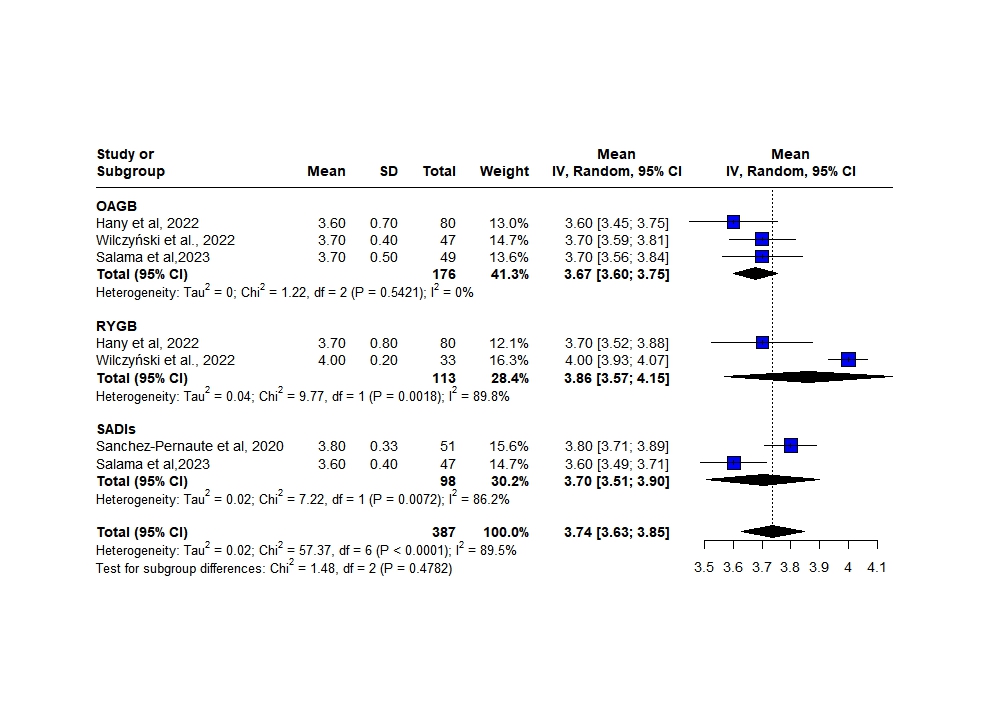


##

## Figure S9 Anemia Sensitivity analysis


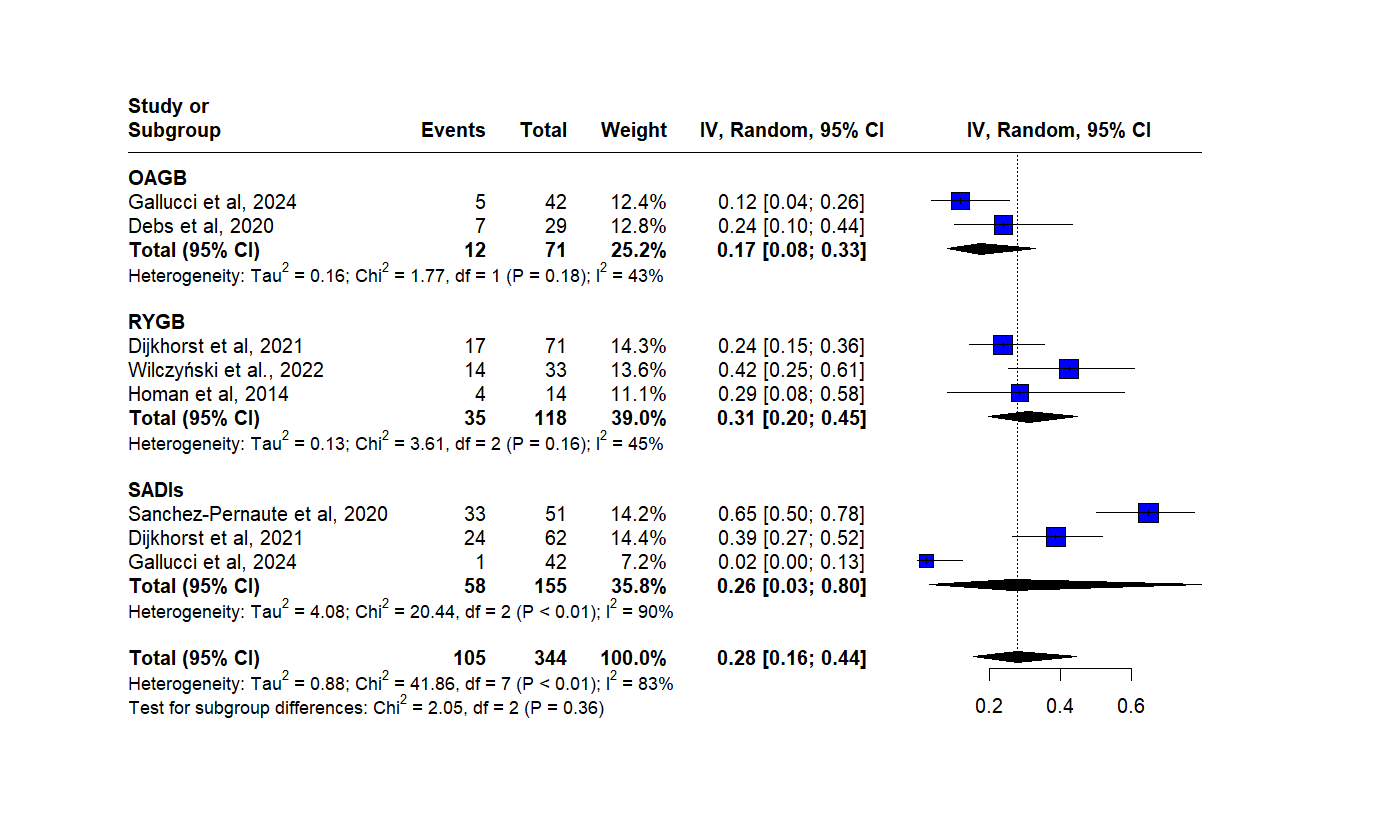


## Figure S10 subgroup of Iron deficiency for OAGB and SADI-S


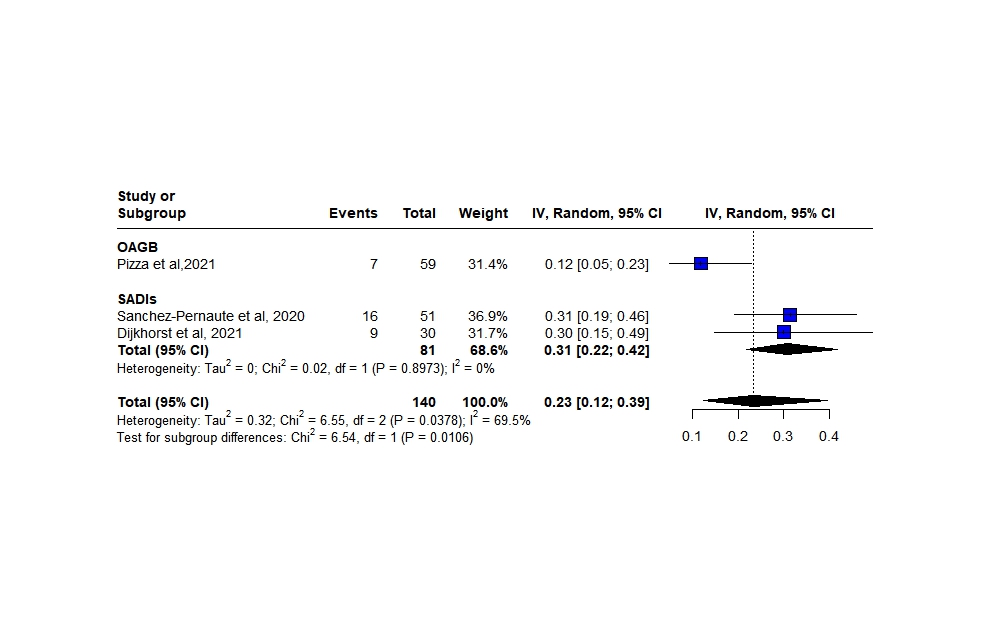


## Figure S11 analysis of ferritin deficiency


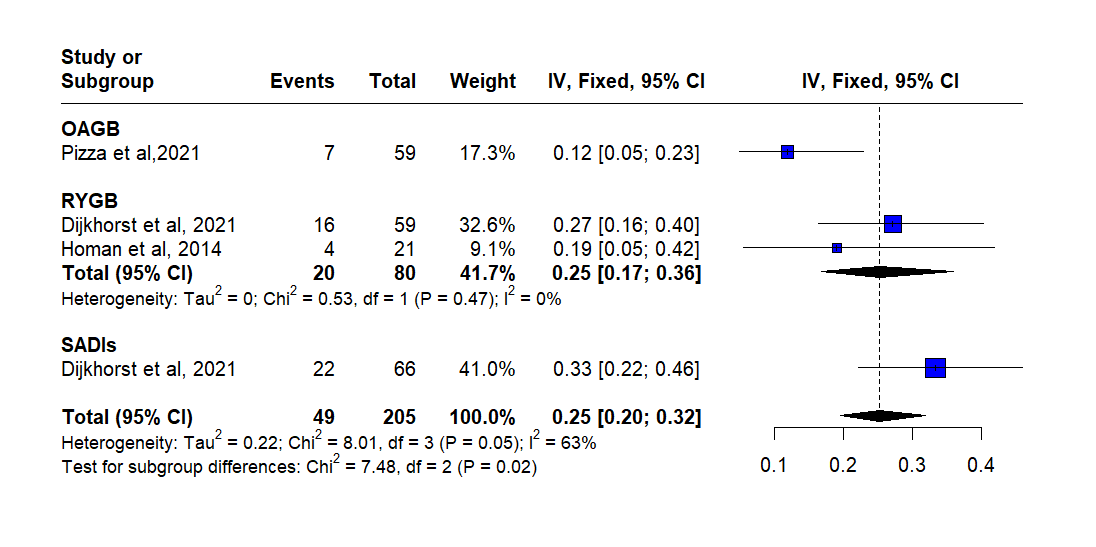


## Figure S12 Zinc deficiency analysis


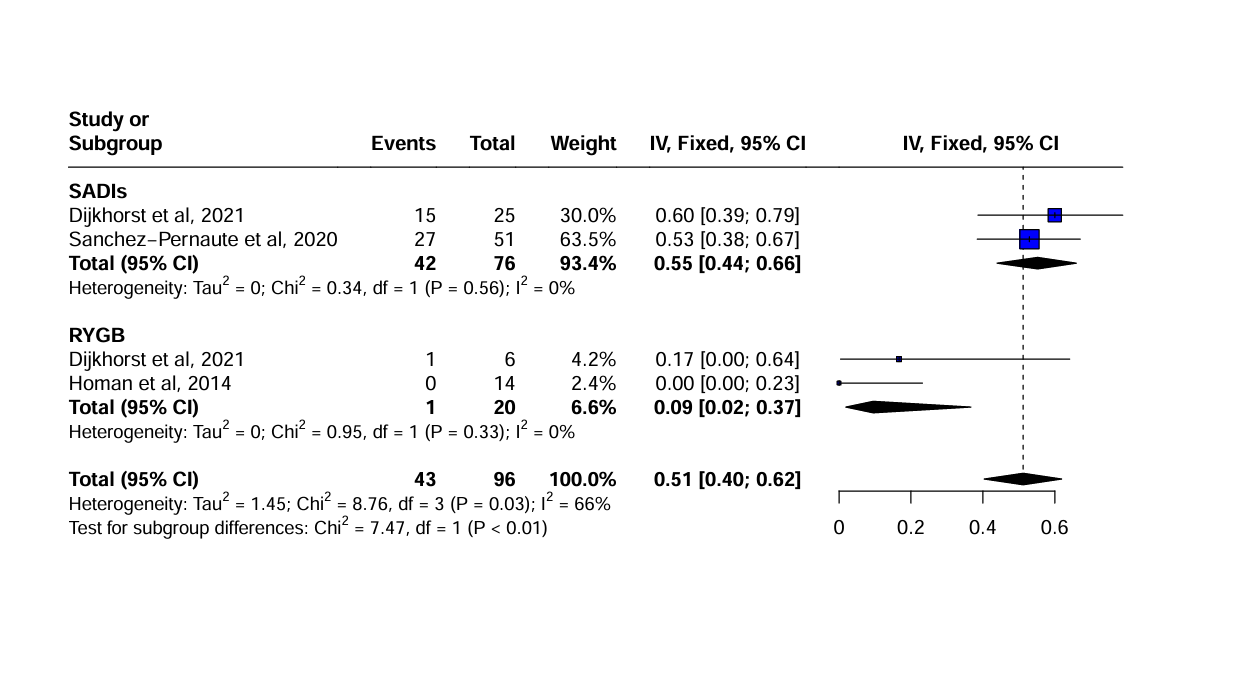

Supplement: Supplementary file 1 — (DOCX 1.67 MB) [file 11695_2025_8325_MOESM1_ESM.docx]
